# Depressive symptoms in long term care facilities in Western Canada: a cross sectional study

**DOI:** 10.1186/s12877-019-1298-5

**Published:** 2019-12-02

**Authors:** Matthias Hoben, Abigail Heninger, Jayna Holroyd-Leduc, Jennifer Knopp-Sihota, Carole Estabrooks, Zahra Goodarzi

**Affiliations:** 1 Faculty of Nursing, University of Alberta, Edmonton, Canada; 2 Faculty of Science, University of British Columbia, Vancouver, Canada; 3 Department of Community Health Sciences, University of Calgary, Calgary, Canada; 4 Hotchkiss Brain Institute, University of Calgary, Calgary, AB Canada; 5 O’Brien Institute of Public Health, Calgary, AB Canada; 6 Cumming School of Medicine, University of Calgary, Calgary, AB Canada; 7 Faculty of Health Disciplines, Athabasca University, Athabasca, Canada

**Keywords:** Depression, Cognitive impairment, Long term care, Inter-RAI

## Abstract

**Background:**

The main objective is to better understand the prevalence of depressive symptoms, in long-term care (LTC) residents with or without cognitive impairment across Western Canada. Secondary objectives are to examine comorbidities and other factors associated with of depressive symptoms, and treatments used in LTC.

**Methods:**

11,445 residents across a random sample of 91 LTC facilities, from 09/2014 to 05/2015, were stratified by owner-operator model (private for-profit, public or voluntary not-for-profit), size (small: < 80 beds, medium: 80–120 beds, large > 120 beds), location (Calgary and Edmonton Health Zones, Alberta; Fraser and Interior Health Regions, British Columbia; Winnipeg Health Region, Manitoba).

Random intercept generalized linear mixed models with depressive symptoms as the dependent variable, cognitive impairment as primary independent variable, and resident, care unit and facility characteristics as covariates were used. Resident variables came from the Resident Assessment Instrument – Minimum Data Set (RAI-MDS) 2.0 records (the RAI-MDS version routinely collected in Western Canadian LTC). Care unit and facility variables came from surveys completed with care unit or facility managers.

**Results:**

Depressive symptoms affects 27.1% of all LTC residents and 23.3% of LTC resident have both, depressive symptoms and cognitive impairment. Hypertension, urinary and fecal incontinence were the most common comorbidities. Cognitive impairment increases the risk for depressive symptoms (adjusted odds ratio 1.65 [95% confidence interval 1.43; 1.90]). Pain, anxiety and pulmonary disorders were also significantly associated with depressive symptoms. Pharmacologic therapies were commonly used in those with depressive symptoms, however there was minimal use of non-pharmacologic management.

**Conclusions:**

Depressive symptoms are common in LTC residents –particularly in those with cognitive impairment. Depressive symptoms are an important target for clinical intervention and further research to reduce the burden of these illnesses.

## Background

Residents of long term care (LTC) facilities are often frail with multiple comorbidities, poor physical function, cognitive impairment and in many cases concomitant depression [1, 2]. It is estimated in Canada, that up to 44% of those living in LTC have depression [3]. Those living in LTC suffer reduced quality of life [3] and poor function [3] when they have co-morbid depression. Interestingly, the burden of depression is not specific to those who meet solely diagnostic criteria, as those with clinical symptoms also have poor quality of life [3]. Prevalence estimates may be conservative, as evidence suggests that depression is under-diagnosed [3] in LTC.

Depression frequently co-occurs with dementia [4]. In comparison to cognitively intact adults, those with dementia have over two times the risk of developing depression (odds ratio (OR) of 2.64 (95% confidence interval (CI) 2.43; 2.86)) [4]. Existing observational data suggest that depression may be a risk factor for dementia, however depressive symptoms can also be early symptoms of dementia [5]. Residents in LTC commonly experience dementia, given this understanding depression as a comorbidity is important [6]. There are available tools to detect depression in LTC residents [7, 8]; however, use of these tools is limited due to numerous barriers contributing to challenges in detection [9]. There are available therapies for depression in those with and without dementia [10–13]. There are several risk factors for depression in LTC, the most commonly studied are cognitive impairment, functional disability and baseline depression [14]. However few studies that examine psychological, environmental factors [14].

Depression in LTC residents and in those with dementia is a target for research aimed at understanding this disease in context, to better target resources and improve diagnosis and treatment. A recent systematic review identified several studies examining the prevalence of depression in LTC, however reported no studies within the Canadian context [15]. The reported range of depression was 5–25% for major depression and 14–82% for depressive symptoms in these studies [15]. We were able to identify a Canadian Institute for Health Information (CIHI) report on depression in LTC, however this was focused only on Ontario, Nova Scotia, Manitoba, Saskatchewan and the Yukon [3]. This CIHI report focuses on depressive symptoms as measured by the Depression Rating Scale collected on the interRAI Resident Assessment Instrument Minimum Data Set, Version 2.0 from the Continuing Care Reporting System [3, 16]. They demonstrated that depressive symptoms were present in 44% of participants, with 26% having a depression diagnosis (*n* = 49,089) [3]. More evidence is needed examining the prevalence of depression in LTC in the western Canadian provinces. It is also unclear in the existing literature how the unit and facility level factors impact depression on the larger scale. It is crucial to understand how depression affects persons living in LTC across Canada in order to inform policy development.

Our primary objectives are to (a) determine the current prevalence of depressive symptoms in LTC residents using cross sectional data across three western provinces, (b) and to understand how this prevalence differs with and without cognitive impairment.. Our secondary objectives were to (a) explore the relationship between depressive symptoms and other prevalent co-morbidities, (b) identify individual and facility factors, and to (c) examine the association of depressive symptoms with available pharmacologic and non-pharmacologic treatments.

## Methods

### Ethics

Ethics approval was obtained for this study from the appropriate university bodies. Ethics approval was obtained for this study from the University of Calgary (CHREB17–0776) and prior approval for the data collection from University of Alberta (PRO00037937) University of British Columbia (H14–00942), and University of Manitoba (H24014:370(HS17856)).

### Study design and setting

This is a cross-sectional analysis of data collected in a representative cohort of 91 urban nursing homes in Western Canada participating in the Translating Research in Elder Care (TREC) program of research [17]. TREC LTC facilities are randomly selected from lists that include all LTC facilities in the participating health regions. Lists are stratified by (a) health region (Calgary and Edmonton Zones in Alberta; Fraser and Interior Health Regions in British Columbia; Winnipeg Region Health Authority in Manitoba), (b) facility size (small, < 80 beds; medium, 80–120 beds; large, > 120 beds), and (c) owner-operator model (private for-profit, public not-for-profit, and voluntary not-for-profit).

### Sample

TREC data include Resident Assessment Instrument – Minimum Data Set 2.0 (RAI-MDS 2.0) [18] data from all residents living in participating nursing homes on a quarterly basis since 2007. While newer versions of this tool are available (e.g., the RAI-MDS 3.0 used in US nursing homes [19] or the i*nterRAI* LTCF in use in one Canadian province [20]) the RAI-MDS 2.0 is the version mandated and routinely collected in all other Canadian provinces (including the five Western Canadian health regions participating in TREC). From this resident data base, we selected a cross-sectional sample of residents that we linked to survey data from facilities, care units and care staff that TREC collects in waves. Care staff data (not used in this study) and care unit and facility characteristics are collected using validated TREC surveys (details reported elsewhere).(17)We used the latest wave of TREC survey data collection (09/2014–05/2015). Of all resident assessments completed in this period, we included each resident’s latest assessment in this period. Our resident sample includes 11,445 nursing home residents living on 325 care units in 91 nursing homes.

### Outcomes and measures

#### Dependent variable

The dependent variable was depressive symptoms, measured with the Depression Rating Scale (DRS) [21]. The DRS is created by summing the scores of seven items: (a) resident made negative statements (passive suicidal ideation), (b) persistent anger with self or others, (c) expressions of what appear to be unrealistic fears, (d) repetitive health complaints, (e) repetitive anxious complaints or concerns, (f) sad, pained, worried facial expressions, (g) crying, tearfulness. Each item can take on the scores of 0 (not exhibited in last 30 days), 1 (exhibited up to 5 days a week), or 2 (exhibited 6 or 7 days a week), leading to a possible range of the DRS of 0–14. US studies [22, 23] found acceptable specificity rates of the DRS (i.e., rate of residents correctly identified as not having depression > 80%) when compared with the Hamilton Depression Rating Scale, [24, 25] the Geriatric Depression Scale, [26, 27] chart reviews, or gold standard clinical assessments by a psychiatrist. However, sensitivity of the DRS was low (i.e., rate of residents correctly identified as having depression < 50%) [22, 23]. A recent review found 9 studies validating the DRS, of these studies most included a percentage of patients with dementia (15–70%), only one focused only on those with dementia [28]. A Canadian study found that the DRS at admission predicts a depression diagnosis at follow-up assessments [29]. The cut off for the DRS is ≥3 for detection symptoms of depression, that are more than moderate [3, 21]. Some recent work has shown that even a score of 1–2 can be predictive of patients developing depression. As a result of these latter two factors we dichotomized the DRS and used a cut-off score of ≥2 to indicate presence of depressive symptoms [29]. Further sensitivity analyses are described below.

#### Primary independent variable

The primary independent variable was cognitive impairment, measured with the RAI-MDS 2.0 Cognitive Performance Scale (CPS) [30]. We preferred the CPS scale over the diagnosis of dementia variables, as dementia is underestimated by at least 11% in the Canadian RAI-MDS 2.0 [31]. Studies have repeatedly confirmed high reliability and validity of the CPS scale [32–34]. We created a dichotomous variable reflecting no cognitive impairment (CPS score < 2) or cognitive impairment of any kind (mild to severe) (CPS score ≥ 2). We chose this cut off to represent symptoms of cognitive impairment and this score has been found to be similar to the MMSE in the detection of cognitive impairment in LTC [35]. We adjusted our statistical models for RAI-MDS 2.0 variables listed in Table 1. These covariates were chosen, as they are relevant conditions that are linked to depression in prior studies. We chose to focus on comorbidities in these individuals as they are clearly defined in the databases and rigorously collected.

**Table 1:** Resident Level Covariates & Justification

Outcome	RAI-MDS 2.0 variable(s)	
*Resident Demographics*		
Age	Calculated as difference between assessment reference date (A3) and birth date (AA3a)	
Sex	AA2	
Marital status	A5	
*Comorbidities*		**Justification for Covariates**
Cardiovascular diseases	Either of arteriosclerotic heart disease (I1d), cardiac dysrhythmia (I1e), congestive heart failure (I1f), deep vein thrombosis (I1g), peripheral vascular disease (I1j), other cardiovascular disease (I1k)	Major depression effects 19% of patients post myocardial infarction^1^. 14 to 60% of patients with heart failure experience depressive symptoms^2^. In peripheral vascular disease between 12 and 24% have depression, however this increases with amputation^3^. A UK study found 18.1% of patients had depressive symptoms^4^. Deep vein thrombosis and post thrombotic syndrome are known to negatively effect health related quality of life^5, 6^.Where DVT was associated with higher anxiety and depression compared to control on the EQ-5D^6^.
Renal failure	I1uu	Across the 5 stages of chronic kidney disease the prevalence of depression 21.4%^7^
Diabetes mellitus	I1a	The relative risk of depression in diabetes is RR 1.27^8^.
Stroke or transient ischemic attack	I1u or I1dd	The prevalence of any depressive disorder in stroke is 33.5%^9^.
Seizure disorder	I1cc	Epilepsy has 22.9% prevalence of depressive disorders^10^
Neurodegenerative disease	Either of amyotrophic lateral sclerosis (I1q), Huntington’s chorea (I1x), multiple sclerosis (I1y), or Parkinson’s disease (I1aa)	In Parkinson’s disease, 35% experience clinically relevant depressive symptoms^11^. For Multiple Sclerosis 30.5% have depression^12^. Those with Amyotrophic Lateral Sclerosis have a OR of depression of 1.7^13^. Approximately 31.7% of those with Huntington’s disease experience major depression^14^.
Traumatic brain injury	I1ee	Traumatic brain injury has a 43% prevalence of depressive disorders^15^
Anxiety disorder	I1ff	Anxiety is common in LTC, with 29.7% of patients reporting anxiety symptoms^16^.
Bipolar disorder	I1hh	Bipolar disorder^17^ includes depressive symptoms as part of the diagnosis
Schizophrenia	I1ii	Depressive symptoms are common (~ 7–75%) patients with schizophrenia^18, 19^, with depression also being part of the diagnostic criteria for schizoaffective disorders^17^.
Cancer	I1rr	8–24% of Cancer patients experience depression^20^.
Respiratory disease	Asthma (I1jj) or emphysema/chronic obstructive pulmonary disease (I1kk)	Pulmonary diseases have been associated with depression^21, 22^ and depression in LTC^23^.
Gastrointestinal disease	I1ss	21.6% of Inflammatory bowel disease patients experience symptoms of depression^24^.
Liver disease	I1tt	Liver diseases, for e.g. non-alcoholic cirrhosis, has an incidence risk ratio for depression of 1.76.^25^
*Other impairments*		
Physical dependency	Activities of Daily Living – Hierarchical^26^ score > 3	Depression is associated with a decline in function (e.g. poor self sufficiency)^27^
Visual impairment	Either of cataracts (I1ll), diabetic retinopathy (I1mm), glaucoma (I1nn), or macular degeneration (I1oo)	Poor vision in seniors is associated with an 1.94 odds of depression (95% CI1.68, 2.25)^28^
Hearing impairment	C1 = 2 (hears in special situations only) or C1 = 3 (hearing highly impaired)	Loss of hearing is associated with depression, OR 1.71 (95%CI 1.28,2.27)^28^.
Pain	Either J2a = 2 (daily pain) or J2b = 3 (phases of excruciating pain regardless of frequency)	Pain and depression are highly correlated across multiple settings^29^.
Outcome	**TREC survey variable**	**Justification for covariates**
Unit type	Care units are either general long term care, non secure dementia, secure dementia, secure mental health/ psychiatric, or other	Our research has demonstrated that quality issues within LTC facilities vary substantially among care units and that unit-level measurement in addition to facility0level measurement is crucial to account for this variance.^30^
Unit staffing	For each care unit TREC collects information on care staffing by care provider group that allows to calculate the care hours per resident day for care aides, licensed practical nurses and registered nurses.^31^	Systematic reviews suggested a link between higher staffing levels and better quality of care (including detection and management of depressive symptoms).^32–34^
Facility location	Facility is located in either the Edmonton or Calgary Health Zone, in the Fraser or Interior Health Authority, or in the Winnipeg Regional Health Authority	The Canadian Health Act requires public payment only for medical services provided in hospitals or by physicians.^35^ Provinces/territories determine individually which services are paid publicly (and how much is paid) and which services clients must cover themselves. Policies regulating LTC differ substantially among Canadian provinces, and so do quality of care issues.^36^ Therefore, and because this is one of the stratification variables to sample TREC facilities, we adjusted our models for facility location.
Facility size	Facility is small (< 80 beds), medium (80–120 beds) or large (> 120 beds)	Evidence suggests that an LTC facility’s size affects quality of care.^37^ Therefore, we adjusted our models for facility location. Therefore, and because this is one of the stratification variables to sample TREC facilities, we adjusted our models for facility location.
Facility owner-operator model	Facility owner operator model is either public not-for-profit, voluntary not-for-profit (e.g., faith based) or private for-profit	Evidence suggests that an LTC facility’s ownership model affects quality of care.^37^ Therefore, we adjusted our models for facility location. Therefore, and because this is one of the stratification variables to sample TREC facilities, we adjusted our models for facility location.
Mental health/geriatric services provided in facility	TREC collects data on whether or not mental health and geriatric services are available in each TREC facility. Services include geriatric mental health consulting, geriatrician, psychiatrist or geriatric psychiatrist, each coded as 1 (available) or 0 (not available)	Availability of mental health services is key to detection and appropriate management of depressive symptoms in older adults^38^.

1. Forrester, AW, Lipsey, JR, Teitelbaum, ML, et al. Depression following myocardial infarction. Int J Psychiatry Med 1992;22 (1):33–46

2. Delville, CL, McDougall, G. A systematic review of depression in adults with heart failure: instruments and incidence. Issues Ment Health Nurs 2008;29 (9):1002–1017

3. Pratt, AG, Norris, ER, Kaufmann, M. Peripheral vascular disease and depression. J Vasc Nurs 2005;23 (4):123–127; quiz 128–129

4. Ismail, H, Coulton, S. Arrhythmia care co-ordinators: Their impact on anxiety and depression, readmissions and health service costs. Eur J Cardiovasc Nurs 2016;15 (5):355–362

5. Kahn, SR, Hirsch, A, Shrier, I. Effect of postthrombotic syndrome on health-related quality of life after deep venous thrombosis. Arch Intern Med 2002;162 (10):1144–1148

6. Utne, KK, Tavoly, M, Wik, HS, et al. Health-related quality of life after deep vein thrombosis. Springerplus 2016;5 [1]:1278

7. Palmer, S, Vecchio, M, Craig, JC, et al. Prevalence of depression in chronic kidney disease: systematic review and meta-analysis of observational studies. Kidney Int 2013;84 (1):179–191

8. Hasan, SS, Mamun, AA, Clavarino, AM, et al. Incidence and risk of depression associated with diabetes in adults: evidence from longitudinal studies. Community Ment Health J 2015;51 (2):204–210

9. Mitchell, AJ, Sheth, B, Gill, J, et al. Prevalence and predictors of post-stroke mood disorders: A meta-analysis and meta-regression of depression, anxiety and adjustment disorder. Gen Hosp Psychiatry 2017;47:48–60

10. Scott, AJ, Sharpe, L, Hunt, C, et al. Anxiety and depressive disorders in people with epilepsy: A meta-analysis. Epilepsia 2017;58 (6):973–982

11. Reijnders, JS, Ehrt, U, Weber, WE, et al. A systematic review of prevalence studies of depression in Parkinson’s disease. Mov Disord 2008;23 (2):183–189; quiz 313

12. Boeschoten, RE, Braamse, AMJ, Beekman, ATF, et al. Prevalence of depression and anxiety in Multiple Sclerosis: A systematic review and meta-analysis. J Neurol Sci 2017;372:331–341

13. Roos, E, Mariosa, D, Ingre, C, et al. Depression in amyotrophic lateral sclerosis. Neurology 2016;86 [24]:2271–2277

14. Slaughter, JR, Martens, MP, Slaughter, KA. Depression and Huntington’s disease: prevalence, clinical manifestations, etiology, and treatment. CNS Spectr 2001;6 (4):306–326

15. Scholten, AC, Haagsma, JA, Cnossen, MC, et al. Prevalence of and Risk Factors for Anxiety and Depressive Disorders after Traumatic Brain Injury: A Systematic Review. J Neurotrauma 2016;33 (22):1969–1994

16. Smalbrugge, M, Pot, AM, Jongenelis, K, et al. Prevalence and correlates of anxiety among nursing home patients. J Affect Disord 2005;88 (2):145–153

17. American_Psychiatric_Association. Diagnostic and statistical manual of mental disorders: DSM-5. Washington, D.C: American Psychiatric Association, 2013

18. Hasan, A, Falkai, P, Wobrock, T, et al. World Federation of Societies of Biological Psychiatry (WFSBP) Guidelines for Biological Treatment of Schizophrenia. Part 3: Update 2015 Management of special circumstances: Depression, Suicidality, substance use disorders and pregnancy and lactation. The world journal of biological psychiatry: the official journal of the World Federation of Societies of Biological Psychiatry 2015;16 (3):142–170

19. Gregory, A, Mallikarjun, P, Upthegrove, R. Treatment of depression in schizophrenia: systematic review and meta-analysis. Br J Psychiatry 2017;211 (4):198–204

20. Krebber, AM, Buffart, LM, Kleijn, G, et al. Prevalence of depression in cancer patients: a meta-analysis of diagnostic interviews and self-report instruments. Psychooncology 2014;23 (2):121–130

21. Bozek, A, Rogala, B, Bednarski, P. Asthma, COPD and comorbidities in elderly people. J Asthma 2016;53 [9]:943–947

22. Matte, DL, Pizzichini, MM, Hoepers, AT, et al. Prevalence of depression in COPD: A systematic review and meta-analysis of controlled studies. Respir Med 2016;117:154–161

23. Barca, ML, Selbaek, G, Laks, J, et al. Factors associated with depression in Norwegian nursing homes. Int J Geriatr Psychiatry 2009;24 (4):417–425

24. Neuendorf, R, Harding, A, Stello, N, et al. Depression and anxiety in patients with Inflammatory Bowel Disease: A systematic review. J Psychosom Res 2016;87:70–80

25. Perng, CL, Shen, CC, Hu, LY, et al. Risk of depressive disorder following non-alcoholic cirrhosis: a nationwide population-based study. PLoS One 2014;9 (2):e88721

26. Morris, JN, Fries, BE, Morris, SA. Scaling ADLs within the MDS. Journals of Gerontology Series A, Biological Sciences and Medical Sciences 1999;54 (11):M546-M553

27. Canadian_Institute_for_Health_Information. Depression Among Seniors in Residential Care. 2010

28. Huang, CQ, Dong, BR, Lu, ZC, et al. Chronic diseases and risk for depression in old age: a meta-analysis of published literature. Ageing Res Rev. 2010;9 (2):131–141

29. Bair, MJ, Robinson, RL, Katon, W, et al. Depression and pain comorbidity: a literature review. Arch Intern Med 2003;163 [20]:2433–2445

30. Norton, PG, Murray, M, Doupe, MB, et al. Facility versus unit level reporting of quality indicators in nursing homes when performance monitoring is the goal. BMJ Open 2014;4 (2):e004488

31. Cummings, GG, Doupe, M, Ginsburg, L, et al. Development and Validation of A Scheduled Shifts Staffing (ASSiST) Measure of Unit-Level Staffing in Nursing Homes. Gerontologist 2017;57 (3):509–516

32. Bostick, JE, Rantz, MJ, Flesner, MK, et al. Systematic review of studies of staffing and quality in nursing homes. Journal of the American Medical Directors Association 2006;7 (6):366–376

33. Castle, NG. Nursing home caregiver staffing levels and quality of care - A literature review. J Appl Gerontol 2008;27 [4]:375–405

34. Spilsbury, K, Hewitt, C, Stirk, L, et al. The relationship between nurse staffing and quality of care in nursing homes: a systematic review. Int J Nurs Stud 2011;48 (6):732–750

35. Deber, R, B., Laporte, A. Funding long-term care in Canada: Who is responsible for what? HealthcarePapers 2016;15 [4]:36–40

36. Health Canada. Long-term facilities-based care; https://www.canada.ca/en/health-canada/services/home-continuing-care/long-term-facilities-based-care.html. Accessed 2017-04-06

37. Tanuseputro, P, Chalifoux, M, Bennett, C, et al. Hospitalization and Mortality Rates in Long-Term Care Facilities: Does For-Profit Status Matter? J Am Med Dir Assoc 2015;16 (10):874–883

38. MacCourt, P, Wilson, K, Tourigny-Rivard, M-F. Guidelines for Comprehensive Mental Health Services for Older Adults in Canada. Calgary, Alberta: Mental Health Commission of Canada; 2011

In addition to covariates (Table 1) included in our statistical models, we assessed use of the following medications in residents with depressive symptoms: antidepressants, antipsychotics, anti-anxiety medication (interRAI data). Looking at only residents with depressive symptoms we assessed the use of antidepressants, antipsychotics, anti-anxiety and pain medications. This was to see what medications those with depressive symptoms were prescribed. However, this has some limitations, as patients who are appropriately treated for depression may not have symptoms and thus not be detected here [36], additionally we cannot account for those started on antidepressants for other indications [37]. Finally, we assessed the following non-pharmacological treatments in residents with depressive symptoms: psychological therapy, special behavior symptom evaluation program, evaluation by a licensed mental health specialist in last 90 days, group therapy, resident-specific deliberate changes in environment, and reorientation.

#### Unit-level covariates

We included the unit type as measured by our TREC unit survey. Units are categorized as either general long-term care, non-secure dementia, secure dementia, secure mental health/psychiatric, or other. We also added measures for staffing hours per resident day on each unit. We included separate measures for care aide, licensed practical nurse (LPN) and registered nurse (RN) hours per resident day [38].

#### Facility-level covariates

Facility location (health region), size, and owner-operator model were included as covariates (TREC Survey Data). Three dichotomous variables were added, indicating whether or not care was provided by a geriatrician, a psychiatrist, or a geriatric psychiatrist were available in a facility (interRAI data).

### Statistical analyses

We used SAS 9.4® [39] for all analyses. If the included assessment was a quarterly form (and hence certain items that are only include in the full assessment forms were missing), we carried forward the values of these items from the previous full assessment [1]. We calculated means and standard deviations for continuous outcomes and numbers and percentages for dichotomous outcomes for the total sample and by health region. Regional differences for each of the outcomes were assessed, using ANOVA for continuous outcomes that met assumptions of normality and homogeneity of variances and Kruskal-Wallis tests for continuous outcomes that violated these assumptions. Fisher’s Exact tests were used tests for categorical outcomes. In residents with depressive symptoms, we assessed differences between residents with and without cognitive impairment in addition to regional differences, using the same statistical methods.

To assess the association of cognitive impairment and of other covariates with depressive symptoms, a three-level random intercept generalized linear mixed models was run [40]. We used a logit link function due to the dichotomous dependent variable (depressive symptoms present or absent) and accounted for dependencies of assessments collected from residents nested within care units and care units nested within facilities by including random unit- and facility-level intercepts. To assess whether the nested model was statistically significantly differed from a non-nested (one-level) model, we performed a covariance test for model independence [41]. These tests indicated that accounting for the clustered structure of the data was necessary (*p* < 0.0001). We also calculated intra-cluster correlation coefficients for unit- and facility levels (i.e. level-specific variance divided by the total variance). We assessed multicollinearity of model covariates by regressing all model covariates on our depressive symptoms variable, using a multiple linear regression, and specified the collinearity diagnostics (COLL) and variance inflation factor (VIF) options [42]. VIF values ≥10 are commonly considered an indicator that a collinearity problem may be present – although even higher VIF values have been discussed as acceptable [43]. Furthermore, variables with a condition index ≥10 that contribute strongly to the variance of two or more other variables (variance proportion > 0.5) also indicate collinearity problems [44]. Our analyses indicated no multicollinearity problem of our covariates. VIF values ranged between 1.015 (traumatic brain injury) and 4.231 (widowed marital status), and none of the variables explained a variance proportion of > 0.5 of two or more of the other variables. Due to the way RAI-MDS 2.0 data are collected and cleaned in Canada, our data set did not include any missing values. The completeness and integrity of RAI-MDS 2.0 items are extremely high in Canada due to universal use of electronic entry that only allows submission of an assessment when all items are populated with valid values [45]. Furthermore, the Canadian Institute for Health Information, the national agency to which TREC facilities submit RAI-MDS 2.0 data, performs additional data checks on submitted records [45]. Hence, missing items were not an issue in our analyses. We first ran a model with only cognitive impairment included as dependent variable. We then added the other covariates one-by-one in a stepwise approach (see Additional file 1 for parameter estimates of all models). For sensitivity analyses, we ran our final model again (see statistical analyses), and exchanged the dichotomous cognitive impairment variable based on a CPS cut-off ≥2 by another dichotomous variable that indicated cognitive impairment if either (a) the CPS score was ≥2 or (b) the resident had a diagnosis of dementia.

## Results

### Description of sample characteristics (Table 2)

Among the 11,445 residents, 67.8% (*n* = 7762) were female with a mean age of 84.7 (SD 10.2). The majority of residents were widowed (49.9%) or married (25.5%). Overall 40.1% had depressive symptoms (*n* = 4594). Cognitive impairment was the most common comorbidity at 81.6% (*n* = 9333), which was similar across all locations. The proportion of residents with both depressive symptoms and cognitive impairment was 34.8% (*n* = 3987). Several comorbidities had a prevalence of over 50%, including hypertension (53.3%), fecal (54.3%) and urinary incontinence (71.9%). Responsive behaviours were also common at 45.5%. Daily pain affected 10.2% of individuals and 15% had fallen in the past 30 days.

**Table 2:** Description of Sample Characteristics

	Calgary (*n* = 2705)		Edmonton (*n* = 2599)		Fraser (*n* = 2749)		Interior (*n* = 1318)		Winnipeg (*n* = 2074)		P	Total (*n* = 11,445)	
Demographics	M	SD	M	SD	M	SD	M	SD	M	SD		M	SD
Age	84.4	10.2	83.8	11.5	85.0	9.7	85.8	9.8	85.8	9.4	**< 0.0001**^**a**^	84.7	10.2
	**N**	**%**	**N**	**%**	**N**	**%**	**N**	**%**	**N**	**%**		**N**	**%**
Female	1767	65.3	1691	65.1	1888	68.7	866	65.7	1550	74.7	**< 0.0001**^**b**^	7762	67.8
Marital status													
Never married	222	8.2	233	9.0	154	5.6	92	7.0	244	11.8	**< 0.0001**^**b**^	945	8.3
Married	738	27.3	689	26.5	760	27.6	213	16.2	523	25.2		2923	25.5
Widowed	1341	49.6	1223	47.1	1394	50.7	642	48.7	1113	53.7		5713	49.9
Separated	60	2.2	59	2.3	75	2.7	220	16.7	25	1.2		439	3.8
Divorced	292	10.8	176	6.8	278	10.1	137	10.4	161	7.8		1044	9.1
Unknown	52	1.9	219	8.4	88	3.2	14	1.1	8	0.4		381	3.3
Comorbidities													
Depressive symptoms	1102	40.8	922	35.5	382	13.9	375	28.5	314	15.1	**< 0.0001**^**b**^	3095	27.1
Cognitive impairment	2264	83.7	2208	85.0	2178	79.2	1069	81.1	1614	77.8	**< 0.0001**^**b**^	9333	81.6
Depressive symptoms and cognitive impairment	953	35.5	804	30.9	317	11.5	323	24.5	274	13.2	**< 0.0001**^**b**^	2671	23.3
Diabetes mellitus	614	22.7	587	22.6	550	20.0	244	18.5	465	22.4	**0.0031**^**b**^	2460	21.5
Thyroid disease	202	7.5	289	11.1	179	6.5	86	6.5	380	18.3	**< 0.0001**^**b**^	1136	9.9
HTN	1488	55.0	1433	55.1	1338	48.7	612	46.4	1227	59.2	**< 0.0001**^**b**^	6098	53.3
Stroke/TIA	568	21.0	597	23.0	590	21.5	308	23.4	483	23.3	0.1619^b^	2546	22.3
Hemiplegia/hemiparesis	205	7.6	157	6.0	99	3.6	49	3.7	35	1.7	**< 0.0001**^**b**^	545	4.8
Seizure disorder	152	5.6	160	6.2	144	5.2	61	4.6	104	5.0	0.2664^b^	621	5.4
Cardiovascular disease	1039	38.4	1040	40.0	724	26.3	439	33.3	805	38.8	**< 0.0001**^**b**^	4047	35.4
Cancer	222	8.2	283	10.9	122	4.4	41	3.1	227	10.9	**< 0.0001**^**b**^	895	7.8
COPD/asthma	376	13.9	443	17.0	227	8.3	152	11.5	317	15.3	**< 0.0001**^**b**^	1515	13.2
Renal failure	116	4.3	105	4.0	105	3.8	87	6.6	121	5.8	**0.0002**^**b**^	534	4.7
Osteoporosis	225	8.3	295	11.4	183	6.7	72	5.5	280	13.5	**< 0.0001**^**b**^	1055	9.2
Arthritis	583	21.6	550	21.2	390	14.2	263	20.0	702	33.8	**< 0.0001**^**b**^	2488	21.7
Neurodegenerative disease	116	4.3	155	6.0	84	3.1	58	4.4	144	6.9	**< 0.0001**^**b**^	557	4.9
Anxiety	95	3.5	109	4.2	60	2.2	51	3.9	271	13.1	**< 0.0001**^**b**^	586	5.1
Bipolar	46	1.7	61	2.3	37	1.3	22	1.7	41	2.0	0.0908^b^	207	1.8
Schizophrenia	90	3.3	74	2.8	48	1.7	25	1.9	75	3.6	**< 0.0001**^**b**^	312	2.7
Visual impairment	380	14.0	544	20.9	375	13.6	142	10.8	288	13.9	**< 0.0001**^**b**^	1729	15.1
Gastrointestinal disease	740	27.4	1017	39.1	181	6.6	150	11.4	297	14.3	**< 0.0001**^**b**^	2385	20.8
Liver disease	31	1.1	26	1.0	16	0.6	16	1.2	14	0.7	0.0848^b^	103	0.9
Fecal incontinence	1572	58.1	1926	74.1	1250	45.5	525	39.8	941	45.4	**< 0.0001**^**b**^	6214	54.3
Urinary incontinence	2043	75.5	2216	85.3	1733	63.0	872	66.2	1363	65.7	**< 0.0001**^**b**^	8227	71.9
Indwelling catheter	137	5.1	174	6.7	87	3.2	72	5.5	75	3.6	**< 0.0001**^**b**^	545	4.8
Responsive behaviors	1362	50.4	1434	55.2	1050	38.2	582	44.2	778	37.5	**< 0.0001**^**b**^	5206	45.5
Fell in past 30 days	428	15.8	392	15.1	373	13.6	210	15.9	313	15.1	0.1405^b^	1716	15.0
Stag 2+ pressure ulcer	157	5.8	200	7.7	119	4.3	50	3.8	65	3.1	**< 0.0001**^**b**^	591	5.2
Stage 2+ stasis ulcer	157	5.8	200	7.7	119	4.3	50	3.8	65	3.1	**< 0.0001**^**b**^	591	5.2
Hip fracture in last 180 days	48	1.8	42	1.6	23	0.8	10	0.8	18	0.9	**0.0015**^**b**^	141	1.2
Traumatic brain injury	63	2.3	78	3.0	56	2.0	36	2.7	25	1.2	**< 0.0001**^**b**^	258	2.3
Aphasia	172	6.4	329	12.7	91	3.3	30	2.3	34	1.6	**< 0.0001**^**b**^	656	5.7
Daily or excruciating pain	179	6.6	196	7.5	345	12.6	188	14.3	258	12.4	**< 0.0001**^**b**^	1166	10.2

^a^*P* value is based on an Analysis of Variance (ANOVA)

^b^*P* value is based on a Fisher’s Exact test

Bold entries is meant to indicate where the *p* value is significant

### Description of LTC facilities (Table 3)

Among the 91 facilities, most facilities were in the Fraser region (*n* = 27) and fewest in the interior of British Columbia and Calgary (*n* = 15 each). Majority of facilities were large (> 120 beds; *n* = 38). Of 91 facilities (*n* = 42) were private for-profit. All sites had access to geriatric mental health counselling services, but access to geriatricians, geriatric psychiatrists and psychiatrists was variable. Most units were general LTC (68%; *n* = 220) or secure dementia units (18.2%; *n* = 59). Care aids, the major provider of direct care, provide a mean of 2.2 h of care per resident per day.

**Table 3:** Description of LTC Facilities

Care facilities													
	Calgary (*n* = 15)		Edmonton (*n* = 18)		Fraser (*n* = 27)		Interior (*n* = 15)		Winnipeg (*n* = 16)		P	Total (*n* = 91)	
	*N*	%	*N*	%	N	%	N	%	N	%		N	%
Size													
Small (< 80 beds)	4	26.7	3	16.7	7	25.9	5	33.3	2	12.5	**0.0142**^**a**^	21	23.1
Medium (80–120 beds)	1	6.7	4	22.2	13	48.1	8	53.3	6	37.5		32	35.2
Large (> 120 beds)	10	66.7	11	61.1	7	25.9	2	13.3	8	50.0		38	41.8
Owner-operator model													
Private for-profit	7	46.7	7	38.9	15	55.6	7	46.7	6	37.5	0.2459^a^	42	46.2
Public not-for-profit	3	20.0	3	16.7	4	14.8	6	40.0	1	6.3		17	18.7
Voluntary not-for-profit	5	33.3	8	44.4	8	29.6	2	13.3	9	56.3		32	35.2
Mental health/geriatric services													
Geriatric mental health consulting	15	100.0	18	100.0	27	100.0	15	100.0	16	100.0	NA	91	100.0
Geriatrician	8	53.3	13	72.2	18	66.7	8	53.3	10	62.5	0.5704^a^	56	61.5
Psychiatrist	8	53.3	17	94.4	21	77.8	10	66.7	12	75.0	0.0810^a^	68	74.7
Geriatric psychiatrist	8	53.3	16	88.9	24	88.9	13	86.7	15	93.8	**0.0339**^**a**^	76	83.5
**Care units**													
	**Calgary (*n*** **= 62)**		**Edmonton (*n*** **= 60)**		**Fraser (n = 91)**		**Interior (*n*** **= 53)**		**Winnipeg (n = 59)**		**P**	**Total (*n*** **= 325)**	
	**N**	**%**	**N**	**%**	**N**	**%**	**N**	**%**	**N**	**%**		**N**	**%**
Unit type													
General long term care	38	61.3	39	65.0	69	75.8	21	39.6	54	91.5	**< 0.0001**^**a**^	221	68.0
Non secure dementia	1	1.6	6	10.0	3	3.3	2	3.8	0	0.0		12	3.7
Secure dementia	19	30.6	9	15.0	15	16.5	11	20.8	5	8.5		59	18.2
Secure mental health/psychiatric	1	1.6	1	1.7	1	1.1	0	0.0	0	0.0		3	0.9
Other	3	4.8	5	8.3	3	3.3	19	35.8	0	0.0		30	9.2
	**M**	**SD**	**M**	**SD**	**M**	**SD**	**M**	**SD**	**M**	**SD**		**M**	**SD**
Staffing hours/resident day													
Care aides	2.3	0.9	2.5	0.7	2.0	0.5	2.1	0.4	2.1	0.3	**< 0.0001**^**b**^	2.2	0.7
Licensed practical nurses	0.6	0.4	0.7	0.6	0.7	0.5	0.5	0.2	0.5	0.2	0.3875^b^	0.6	0.4
Registered nurses	0.5	0.6	0.4	0.3	0.4	0.3	0.2	0.3	0.4	0.2	**< 0.0001**^**b**^	0.4	0.4

^a^*P* value is based on a Fisher’s Exact test

^b^*P* value is based on a Kruskal-Wallis test

Bold entries is meant to indicate where the *p* value is significant

### Pharmacologic and non-pharmacologic treatment for those with depressive symptoms (Table 4)

When examining the 3095 residents with depressive symptoms, 86.3% (*n* = 2671) had cognitive impairment.

**Table 4:** Pharmacologic and Non-Pharmacologic treatment for those with depressive symptoms

	Cognitive impairment					Health region												
	No		Yes			Calgary Zone		Edmonton Zone		Fraser Health		Interior Health		Winnipeg Health			Total	
	N	%	N	%	P^a^	N	%	N	%	N	%	N	%	N	%	P^a^	N	%
Overall sample of residents with depressive symptoms*	424	13.7	2671	86.3	**< 0.0001**	1102	35.1	922	29.8	382	12.3	375	12.1	314	10.2	**< 0.0001**	3095	100.0
Use of antidepressants																		
1–6 days in last week	2	0.5	26	1.0	0.1478	9	0.8	5	0.5	9	2.4	3	0.8	2	0.6	0.0892	28	0.9
7 days in last week	231	54.6	1566	58.8		639	58.1	551	59.8	217	57.1	224	60.1	166	52.9		1797	58.2
No antidepressants with a diagnosis of depression	35	8.3	182	6.8	0.3052	68	6.2	82	8.9	17	4.5	25	6.7	25	8.0	**0.0342**	217	7.0
Use of antipsychotics**																		
1–6 days in last week	4	0.9	67	2.5	**< 0.0001**	21	1.9	25	2.7	14	3.7	9	2.4	2	0.6	**< 0.0001**	71	2.3
7 days in last week	84	19.9	895	33.6		324	29.5	242	26.3	120	31.6	166	44.5	127	40.4		979	31.7
Antipsychotic use with no diagnosis of psychosis	63	14.9	777	29.2	**< 0.0001**	278	25.3	203	22.0	115	30.3	141	37.8	103	32.8	**< 0.0001**	840	27.2
Use of antianxieties**																		
1–6 days in last week	9	2.1	107	4.0	**< 0.0001**	18	1.6	42	4.6	27	7.1	24	6.4	5	1.6	**< 0.0001**	116	3.8
7 days in last week	84	19.9	328	12.3		100	9.1	144	15.6	67	17.6	58	15.5	43	13.7		412	13.3
No antianxieties with a diagnosis of anxiety	22	5.2	114	4.3	0.3727	30	2.7	38	4.1	8	2.1	14	3.8	46	14.6	**< 0.0001**	136	4.4
No analgesics with pain**	13	3.1	47	1.8	0.0853	34	3.1	14	1.5	5	1.3	5	1.3	2	0.6	**0.0192**	60	1.9
Non-pharmacological treatments**																		
Psychological therapy	1	0.2	21	0.8	0.3480	17	1.5	3	0.3	1	0.3	1	0.3	0	0.0	**0.0043**		
Special behaviour symptom evaluation program	126	29.8	661	24.8	0.1012	324	29.5	220	23.9	100	26.3	106	28.4	37	11.8	**< 0.0001**	787	25.5
Licensed mental health specialist evaluation in last 90 days	25	5.9	97	3.6	**0.0312**	71	6.5	24	2.6	7	1.8	11	2.9	9	2.9	**< 0.0001**	122	4.0
Group therapy	20	4.7	99	3.7	0.3397	67	6.1	28	3.0	10	2.6	7	1.9	7	2.2	**0.0003**	119	3.9
Resident specific deliberate changes in environments	10	2.4	123	4.6	**0.0380**	11	1.0	98	10.6	1	0.3	6	1.6	17	5.4	**< 0.0001**	133	4.3
2003Reorientation	34	8.0	531	19.9	**< 0.0001**	108	9.8	228	24.8	27	7.1	23	6.2	179	57.0	**< 0.0001**	565	18.3

*Percentages are based on overall sample (*n* = 3095 residents with depressive symptoms)

**Percentages are based on total number of residents in the respective column category

^a^P values are based on a Fisher’s Exact test

Bold entries is meant to indicate where the *p* value is significant

Of those who received an antidepressant, 58.2% received antidepressants daily. Of residents with depressive symptoms 7.0% were not on antidepressants. This rate did not differ between residents with and without cognitive impairment. Few residents with depressive symptoms and pain were not receiving analgesics (1.8% in cognitively impaired). Non-pharmacologic strategies were less commonly used. In those with cognitive impairment, behaviour symptom evaluation programs were most commonly used (24.8%), followed by reorientation strategies (19.9%).

### Influence of cognitive impairment and other resident, care unit and facility characteristics on depressive symptoms, based on generalized linear mixed models (Table 5)

Our final model (Table 5) indicates that the odds of experiencing depressive symptoms were almost twice as high in people with cognitive impairment than in people without cognitive impairment. Higher age and female sex also increase the odds for depressive symptoms. Of the assessed comorbidities, only anxiety and respiratory disease were independently associated with depressive symptoms (increased odds, as expected). Of the other impairments pain increased the odds for depressive symptoms and ADL impairment decreased the odds of depressive symptoms. Residents living on secure dementia care units had higher odds of depressive symptoms than residents living on general long-term care units. Odds of depressive symptoms on other unit types did not differ from odds on general long-term care units.

**Table 5:** Influence of cognitive impairment and other resident, care unit and facility characteristics on depressive symptoms, based on generalized linear mixed models

	Model results					
	Est	SE	***P***	OR	95% CI	
Intercept	−2.613	0.308	**< 0.0001**	─	─	─
Cognitive impairment	0.499	0.072	**< 0.0001**	1.648	1.430	1.899
Age	−0.006	0.003	**0.015**	1.006	1.001	1.011
Female	0.386	0.056	**< 0.0001**	1.471	1.318	1.641
Comorbidities						
Anxiety	0.751	0.107	**< 0.0001**	2.119	1.717	2.614
Respiratory disease	0.359	0.069	**< 0.0001**	1.432	1.251	1.639
Other impairments						
Dependency in ADL	−0.111	0.052	**0.033**	0.895	0.809	0.991
Pain	0.980	0.080	**< 0.0001**	2.665	2.278	3.119
Unit type *(ref = general long term care)*						
Non secure dementia	0.331	0.298	0.268	1.392	0.776	2.497
Other	−0.154	0.235	0.514	0.858	0.541	1.360
Secure dementia	0.304	0.143	**0.033**	1.356	1.025	1.793
Secure mental health/psychiatric	0.781	0.512	0.127	2.184	0.800	5.958
Facility location (health region) *ref = Winnipeg Health*						
Calgary Zone	1.648	0.273	**< 0.0001**	5.195	3.040	8.877
Edmonton Zone	1.246	0.266	**< 0.0001**	3.475	2.062	5.857
Fraser Health	0.100	0.248	0.688	1.105	0.680	1.795
Interior Health	0.949	0.297	**0.001**	2.583	1.444	4.620
Facility owner-operator model *(ref = private for-profit)*						
Public not for profit	0.527	0.230	**0.022**	1.693	1.079	2.658
Voluntary not for profit	0.390	0.183	**0.033**	1.476	1.032	2.112
	**Model fit**					
	**Est**					
−2 Log Likelihood	11,114.36					
AICC (smaller is better)	11,154.44					
BIC (smaller is better)	11,204.58					
	**Covariance components**					
	**Est**	**SE**	**P**	**95% CI**		ICC*
Facility	0.333	0.093	0.0002	0.206	0.626	0.092
Unit	0.479	0.070	< 0.0001	0.367	0.650	0.127
	**−2 Res. LL**		**P**			
Test for independence	11,980		< 0.0001			

*Est* Estimate, *SE* Standard Error, *OR* Odds Ratio, *CI* Confidence Interval, *ICC* Intra-cluster Correlation Coefficient

Bold entries is meant to indicate where the *p* value is significant

The model with unit-level variables included (Additional file 1, Model 6) suggested that an increase of care aide hours per resident day decreased the risk for depressive symptoms. However, this variable was no longer significant when facility variables were added (final model, Table 5). Compared to the Winnipeg Health Region, residents living in a nursing home located in the Calgary and Edmonton Health Zones and in the Interior Health Region have a substantially higher odds of depressive symptoms. The odds of depressive symptoms are also higher for residents living in a public or voluntary not-for profit facility, as compared to a private for-profit facility. Facility size and services provided were not statistically significant predictors of depressive symptoms.

## Discussion

Depression in those living in LTC is a complex disease affected by cognitive impairment, multi-morbidity, frailty, and environmental factors. The prevalence of depressive symptoms in LTC is consistently high ranging with a median prevalence of 29% [15]. Our results demonstrate that 27.1% of LTC residents experience depressive symptoms. Nearly 80 % of all LTC residents have cognitive impairment, and of those 23.3% experience depressive symptoms. This estimate furthers our understanding of depression in LTC and what factors may affect these symptoms. This is of critical importance as these other factors may be an important component of developing future intervention studies and management strategies.

Here the DRS is used to measure depressive symptoms. This tool was also used in a 2010 Canadian Institute for Health Information (CIHI) report [3]. This CIHI report found a higher prevalence of depression at 44%, however this examined different regions including Yukon, Saskatchewan, Nova Scotia, Ontario and Manitoba. This report also identifies that cognitive impairment, pain and unstable health conditions are among the common symptoms that effect persons experiencing depressive symptoms’ [3]. Our results identify a lower prevalence of depression, it is possible there is geographic differences in depression. Additionally the analyses presented here are from the 2014–2015 TREC data, where as the CIHI report is from 2008 to 2009 [3]. Interestingly the recent ‘Quick Stats’ CIHI data, which is available online, demonstrates a similar prevalence of depressive symptoms in residential care across to this current analysis multiple provinces 26.2% [16].

Anxiety and pulmonary diseases were independently associated with depressive symptoms. Anxiety is often comorbid with depression in those living in LTC, with 5.1% of cases overlapping (when using strict criteria) [46]. Here, anxiety increased the odds of depression to 2.12 (95%CI 1.72, 2.61). Given anxiety is common in LTC [46] and in those experiencing dementia, [47] this overlap is important from a clinical perspective. Perhaps there should be consideration of screening for both depressive and anxiety symptoms in LTC residents. Of interest, pulmonary diseases were associated with depressive symptoms (1.43; 95% CI 1.25, 1.64). The association of depression and pulmonary disease in LTC was previously noted in other studies [48–50]. This association could be attributable to the symptoms, treatment or prognosis of pulmonary disease, thus additional study is needed.

Pain was independently associated with depressive symptoms (OR 2.67; 95% CI 2.28, 3.12). Similarly another study found that those with pain in LTC are 2.83 times more likely to have prevalent depression [51]. This is a key finding, as the management of residents with depressive symptoms related to pain may need a different approach. However, further research is needed to examine the effectiveness of this treatment approach on both mood and pain, and this approach cannot be recommended based on these results alone.

Of those with depressive symptoms and cognitive impairment, 58.8%, with only 7% of people receiving antidepressants without a diagnosis of depression. Here we examine depressive symptoms and not confirmed depression diagnoses, thus it is expected some residents may not be on treatment. Similarly, persons who are on treatment for depression and not exhibiting depressive symptoms would not be represented in this estimate.

Approximately one third of residents with depressive symptoms and cognitive impairment were receiving antipsychotics for 7 days in the past week. Evidence surrounding the use of antipsychotics in the elderly, specifically those with dementia, suggests increased risk of morbidity and mortality therefore it is important to ensure appropriate use of these drugs [52]. However, this data does not identify the reasons for prescription of antipsychotics, thus we are not able to look at those associations based on these data.

The pharmacologic management of depression is only part of the picture. Non-pharmacologic therapies are also recommended and effective [10]. However, there appeared to be little access to these therapies and not all LTC sites had access to specialty mental health resources. In the CIHI study of depression in residential care, mental health services and non-pharmacologic treatment strategies were also rarely employed [3]. There appears to be a care gap related to the underuse non-pharmacological management. Exploring the lack of availability or use of these services may be key to understanding and developing an approach to improve access.

## Limitations

This study is unique in that we examine a large population of LTC residents in Western Canada, the prevalence of depressive symptoms and explore the association with co-morbidities, facility and treatment factors. In this study, we can only look at associations and not causation, and cannot assert specific conclusions about the effect of diseases on depression or treatment over time. We used the MDS-RAI 2.0 to estimate the prevalence of symptoms, which is a common practice in this population. Although RAI tool administration is standardized and rigorously applied, we cannot control for specific site or unit differences in training, nor the tool accuracy. The DRS has been criticized for its accuracy [28]. This is when examining the accuracy of diagnosing depression, however here we used the DRS to approximate depressive symptoms in residents.

## Conclusions

Depressive symptoms are common in LTC residents. Not surprisingly, cognitive impairment is an independent predictor of depressive symptoms. For those experiencing depressive symptoms, our study has identified several associations with co-morbidities, facility level issues and treatment that warrant in depth study. These represent important targets for future study to both understand and develop better resources to aid in reducing the burden of depression. Understanding that these symptoms are common and the current gaps in related care is key to LTC resource planning.

## Supplementary information

**Additional file 1.**

## Data Availability

These data are not available for public access due to the existing ethics approval and policies of TREC.

## Abbreviations

CIHI: Canadian Institute for Health Information
DRS: Depression Rating Scale.
LTC: Long Term Care
OR: Odds Ratio
RAI-MDS: Resident Assessment Instrument – Minimum Data Set
TREC: Translating Research in Elder Care
